# Evaluation of Indigenous Entomopathogenic Nematodes as Potential Biocontrol Agents against *Popillia japonica* (Coleoptera: Scarabaeidae) in Northern Italy

**DOI:** 10.3390/insects11110804

**Published:** 2020-11-14

**Authors:** Giulia Torrini, Francesco Paoli, Giuseppe Mazza, Stefania Simoncini, Claudia Benvenuti, Agostino Strangi, Eustachio Tarasco, Gian Paolo Barzanti, Giovanni Bosio, Ilaria Cutino, Pio F. Roversi, Leonardo Marianelli

**Affiliations:** 1 CREA Research Centre for Plant Protection and Certification, 50125 Florence, Italy; francesco.paoli@crea.gov.it (F.P.); giuseppe.mazza@crea.gov.it (G.M.); stefania.simoncini@crea.gov.it (S.S.); claudia.benvenuti@crea.gov.it (C.B.); agostino.strangi@crea.gov.it (A.S.); gianpaolo.barzanti@crea.gov.it (G.P.B.); ilaria.cutino@crea.gov.it (I.C.); piofederico.roversi@crea.gov.it (P.F.R.); leonardo.marianelli@crea.gov.it (L.M.); 2 Department of Soil, Plant and Food Sciences, Section of Entomology and Zoology, University of Bari A Moro, 70126 Bari, Italy; eustachio.tarasco@uniba.it; 3 Settore Fitosanitario e Servizi Tecnico-Scientifici—Regione Piemonte, 10144 Torino, Italy; giovanni.bosio@regione.piemonte.it

**Keywords:** alien invasive species, biological control, Heterorhabditidae, Japanese beetle, natural enemies, Steinernematidae

## Abstract

**Simple Summary:**

The Japanese beetle *Popillia japonica* is considered one of the most harmful organisms in the world for crops and the urban landscape. *Popillia japonica* spends most of its life cycle in the soil as a larva. At this stage, this beetle is more susceptible to biological control agents like entomopathogenic nematodes, which are obligate parasites of main soil-inhabiting insects, killing their host in just a few days. In 2014, *P. japonica* was detected in Northern Italy between the Piedmont and Lombardy regions. This research aims to investigate the natural occurrence of indigenous and locally adapted entomopathogenic nematodes along the Piedmont part of the Ticino river and test the most performing of them via bioassays. Natural isolates were recovered from 39 out of the 155 soil samples collected. The virulence of all entomopathogenic nematodes assessed by laboratory and semi-field assays highlighted that two isolates resulted in more efficiency in controlling *P. japonica* grubs. This result is very encouraging, and the use of these natural biocontrol agents against this pest is a fundamental component of eco-friendly management.

**Abstract:**

The natural presence of entomopathogenic nematodes (EPNs) has been investigated in the Piedmont region (Northern Italy) in areas infested by the Japanese beetle *Popillia japonica*. Thirty-nine out of 155 soil samples (25.2%) were positive for EPNs. Most of the samples contained only steinermatids (92.3%), 5.1% contained heterorhabditids, and one sample (2.6%) contained both genera. All the recovered isolates were identified at species level both morphologically and molecularly. *Steinernema carpocapsae* was the most abundant and it was mainly distributed in open habitats, such as perennial meadows, uncultivated soils, and cropland, characterized by sandy loam soil texture and acidic pH. *Steinernema feltiae* has been found associated mainly with closed habitats such as coniferous and deciduous woodland, characterized by sandy loam-texture and extremely acidic soil. The three isolates of *Heterorhabditis bacteriophora* were collected only in open habitats (perennial meadows and uncultivated fields) characterized by strongly acidic soils with sandy loam texture. The virulence of all EPN natural strains was evaluated by laboratory assays against *P. japonica* third-instar larvae collected during two different periods of the year (spring, autumn). The results showed that larval mortality was higher for pre-wintering larvae than post-wintering ones. The five more promising EPN isolates were tested in the semi-field assay in which *H. bacteriophora* natural strains have been shown to be more efficient in controlling *P. japonica* grubs. All of these results are finally discussed considering the use of these natural EPNs as biological control agents against *P. japonica*, within an eco-friendly perspective of management.

## 1. Introduction

The Japanese beetle *Popillia japonica* Newman (Coleoptera: Scarabaeidae) is considered one of the most harmful organisms in the world for agricultural crops and is a key pest for turf, the urban landscape, and ornamental and fruit plants. Native to Japan, *P. japonica* has proven to rapidly spread into new environments due to its ecological plasticity and the ability to feed on more than 300 plant species [1]. In the USA, where *P. japonica* was discovered in 1916 [2], more than USD 460 million is spent annually for the management and mitigation of damage caused by this pest [3].

Mainland Europe has climate and landscapes favorable for *P. japonica* colonization, resulting in damage to crops, pastures, and lawns caused by this beetle [4]. For these reasons and due to this beetle’s ability to be imported via commerce, *P. japonica* has been added to the EU priority pest list (2019/1702/EU Regulation).

In 2014, *P. japonica* was detected in Ticino Natural Park, in Northern Italy, between the Piedmont and Lombardy regions [5]. The infested area has been increasing, reaching, to date, a surface of about 7550 km^2^ [6] with millions of specimens present in the territory [7].

Most of the life cycle of *P. japonica* is spent in the soil as a grub [2]. At this stage, grubs produce serious damages to turf grasses, hayfields, soccer pitches, and golf courses and they are susceptible to biological control agents present in the soil, such as entomopathogenic nematodes (EPNs) and fungi (EPF) [8,9,10]. EPNs are obligate parasites of main soil-inhabiting insects, killing their host in just a few days. Due to this ability, in the last decade, EPNs have been extensively used to control insect pests (e.g., Klein and Kaya [11]; Koppenhöfer et al. [12]). Moreover, EPNs are also an effective control measure against *P. japonica* instead of chemical alternatives [13]. Among the EPNs, Steinernematidae and Heterorhabditidae families exhibit a great variety of adaptations to different abiotic conditions (e.g., soil moisture, texture, pH, temperature) [14,15]. These nematodes are harmless to non-target vertebrates and the environment. Moreover, their effect is self-amplifying and production costs have been significantly reduced in recent times [16].

Ticino Natural Park plays a vital role in preserving biodiversity in one of the most developed and urbanized areas of Europe [17]. However, this natural area was easily and quickly colonized by *P. japonica* due to the favorable edaphic and climatic conditions, such as soil texture and humidity, suitable for its development and spread. Therefore, the invasion of the Japanese beetle might be a threat not only for agriculture but also for the conservation of biodiversity.

Since the Italian infestation of *P. japonica* originally occurred within this park, commercial biological control agents, in the first instance, were tested to cope with this pest with variable results (e.g., Paoli et al. [18]; Marianelli et al. [13]). Indeed, this area showed particularly favorable environmental conditions for the development of entomopathogenic nematode strains.

This fact encourages the search for local and adapted strains of EPNs, but to date, despite the occurrence of a new species of mermithid *Hexamermis popilliae* [19], a profound investigation on the natural occurrence of biological control agents such as EPNs and EPF has been lacking. Thus, the aims of the present contribution were to bridge this knowledge gap by (i) investigating the natural occurrence of indigenous and locally adapted EPNs along the Piedmont part of the Ticino river and (ii) testing the most performing of them via bioassays against *P. japonica* grubs.

The identification of particularly aggressive natural EPN strains would contrast this phytosanitary problem by proposing the use of these natural enemies probably performing better in those specific edaphic conditions.

## 2. Material and Methods

### 2.1. Field Sampling and Isolation of Nematodes

**Figure 1:**
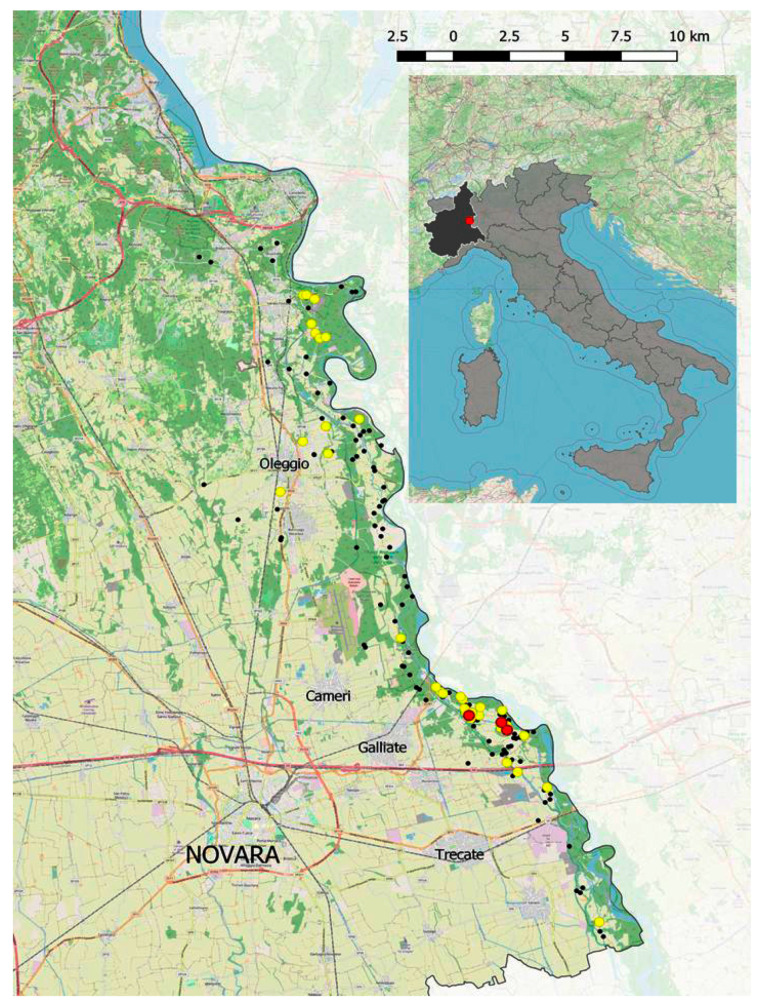
Entomopathogenic nematodes sampling sites map in an area infested by *Popillia japonica* in the Piedmont region (Northern Italy). Black points: sites without nematodes, yellow points: sites with *Steinernema* spp., red points: sites with *Heterorhabditis bacteriophora*.

During the spring of 2017, 155 soil samples were collected from different habitats (meadows, croplands, uncultivated fields, and woodlands) never treated, in previous years, with biocontrol agents. All sampling areas were distributed within an area of approximately 116 km^2^ along the western side of the Ticino river, in Novara province, in the Piedmont region (Northern Italy) (Figure 1).

Each soil sample weighed about 1 kg and resulted from the mixture of five subsamples of about 200 cm^3^ dug with the shovel within an area of 5 × 5 m. All samples were georeferenced in WGS84 (World Geodetic System 1984), labeled as POP 1–155, and before being placed in the plastic bags, *Popillia japonica* larvae were removed from the soil samples and counted. Samples were transferred to the CREA-(Research Centre of Plant Protection and Certification) laboratory of Florence to be processed. Once in the lab, soil samples were placed into plastic containers covered with a perforated lid, and EPNs were recovered by the insect bait method [20]. For each sample, five last-instar *Galleria mellonella* L. (Lepidoptera: Pyralidae) larvae were placed onto the soil and incubated at 23 °C for one week. Soil samples were stirred every day to foster the contact of the bait larvae with the entomopathogenic nematodes present in the sampled soil. Mortality was checked every two days and dead specimens showing signs of EPNs were rinsed in sterile distilled water and placed individually into modified white traps [21] to collect the emerged infective juveniles (IJs). Emerging nematodes were used to infect five *G. mellonella* separate larvae (100 IJs/larva) in Petri dishes (diam. 3.5 cm) to verify pathogenicity, complete Koch’s postulates, and establish the culture.

Nematodes capable of infesting *G. mellonella* larvae for the second time were subsequently collected, stored in single flasks in the darkroom, and incubated at 12 °C. Each flask containing EPNs acquired the same label as the soil from which the contained nematode was isolated (see above). The pH of the soil samples with EPNs was analyzed and correlated with site location, habitat, and soil type, as extracted from Brenna et al. [22] and IPLA [23]

### 2.2. Nematode Identification

Both molecular and morphologic examinations were used for EPN species identification. For morphobiometrical studies, IJs and first-generation males of each isolate were heat-killed in warm water at 65 °C. Then, they were fixed in triethanolamine formalin (TAF) solution (2 cc triethanolamine, 7 cc of 40% commercial formaldehyde solution, and 91 cc distilled water), processed to glycerin by a modified glycerine-ethanol series of the Seinhorst rapid method, and then permanently mounted in anhydrous glycerin on microscope glass slides. Specimens were observed under a light microscope equipped with a Leica MC 170 HD digital camera (Leica Microsystems, Heerbrugg, Switzerland) and the specimens were measured using LEICA Application Suite (LAS) Version 4.9.0 (Leica Microsystems, Heerbrugg, Switzerland).

The features measured for the IJs (*n* = 10 for each EPN) were body length (L), distance from the anterior end to excretory pore (EP), distance from the anterior end to the base of the pharynx (ES), and tail length (T). Moreover, the following ratios were calculated: a (body length/maximum body diameter), b (body length/distance from the anterior end to the base of the pharynx), c (body length/tail length), D% (distance from the anterior end to the excretory pore/distance from the anterior end to the base of the pharynx), and E% (distance from the anterior end to excretory pore/tail length). The IJs’ body length of each species was matched with nematodes that have a similar length in the polytomous key of Nguyen [24], and then other specific characteristics were used to identify the species. Finally, the spicule shape of the males was compared with the original description to confirm the identification.

For the molecular analysis, three specimens of nematodes were collected for each isolate and individually put in a 0.2 mL tube containing 50.0 µL InstaGene Matrix (Bio-Rad, Hercules, CA, USA), 1.5% Sodium Dodecyl Sulfate (SDS), and were frozen at −80 °C overnight. Afterwards, samples were rapidly thawed at 55.0 °C, added to with 2.5 µL Proteinase K (Qiagen, Hilden, Germany) 20.0 µg/µL, and incubated at the same temperature for 3 h. Proteinase K was inactivated by heating at 96.0 °C for 10 min and DNA was recovered by alcoholic precipitation adding 100.0 µL of cold absolute ethanol for each sample. Pellets were air-dried and re-suspended in 20.0 µL of double-distilled water. Amplifications of the internal transcribed spacer locus (ITS) were performed using primers TW81 (5′-GTTTCCGTAGGTGAACCTGC-3′) and AB28 (5′-ATATGCTTAAGTTCAGCGGGT-3′) following a touchdown PCR protocol on 2720 Thermal Cycler (Applied Biosystem, Foster City, CA, USA). Briefly, 5.0 µL of DNA was used in a 50.0 μL amplification reaction containing 1X DreamTaq Hot Start PCR Master Mix (Thermo Fisher Scientific, Waltham, MA, USA) and 0.6 mM of each primer. The thermal protocol adopted was as follows: an initial denaturation/hot start activation step for 3 min at 95 °C followed by 10 cycles of denaturation at 94 °C for 60 s, primers annealing for 60 s from 50 to 45 °C, decrease of −0.5 °C/cycle, elongation at 72 °C for 1.5 min, followed by 35 traditional amplification cycles with denaturation at 94 °C for 60 s, primers annealing for 60 s at 45 °C, elongation at 72 °C for 1.5 min, followed by a final elongation step of 7 min at 72 °C. The PCR products were sequenced at the Centro di Servizi per le Biotecnologie di Interesse Agrario Chimico ed Industriale (CIBIACI), University of Florence, Italy. Sequences were submitted in GenBank. Species attribution was defined with a BLAST similarity search. To solve ambiguities in species attribution inside the *Steinernema* genus, sequences belonging to this species were further characterized by performing a maximum likelihood (ML) phylogenetical reconstruction based on the ITS locus. The ML tree was computed starting from an alignment 1156 positions long, choosing GTR + G + I as the nucleotide substitution model, and validated with 1000 bootstrap pseudo-replicates.

### 2.3. Laboratory Virulence Assays

The experiment was repeated twice, using *Popillia japonica* larvae collected from infested soil of the Piedmont region in May (post-wintering 3rd-instar larvae) and September 2018 (pre-wintering 3rd-instar larvae) following results obtained in Paoli et al. [25]. The grubs were transferred to the CREA-DC laboratory in Florence (Italy) and maintained at 4 °C in native soil. Each test was carried out within 10 days after larval collection. Larvae were acclimated individually in Petri dishes at 20 °C for 4 days before nematode inoculation, to evaluate the absence of preventive infestation by natural EPNs.

Each EPN strain of POP (see above) collected from the positive soil samples (see Results section) was reared at 24 °C in greater wax moth *G. mellonella* late-instar larvae, and the infective juveniles (IJs) were recovered using modified white traps. After storage at 12 °C for a maximum of 2 weeks, they were kept at 20 °C for 24 h before the tests.

Each POP isolate was tested against post-wintering 3rd-instar larvae (*n* = 6) and pre-wintering 3rd-instar larvae of *P. japonica* (*n* = 6). The experimental unit consisted of a Petri dish (3.5 cm diameter) with two layers of filter paper (Whatman No. 1) and one larva. A distilled water suspension of 250 μL containing about 300 IJs (about 31 IJs/cm^2^) was inoculated into each Petri dish. Concerning the mixed isolates, the proportions of the two EPNs were as follows: 45% *S. carpocapsae*/55% *S. feltiae* for POP 138 and 61% *H. bacteriophora*/39% *S. carpocapsae* for POP 80. In the control (*n* = 6), only distilled water was added. Petri dishes were stored at 23 °C in the dark. Larval mortality was checked daily for 14 days. Dead larvae were transferred individually to modified white traps to determine the percentage of emergence (percentage of cadavers from which the progeny emerged) and the time of emergence (i.e., number of days from the death of *P. japonica* larvae to the moment when IJs started to emerge from the infested host cadaver).

### 2.4. Semi-Field Virulence Assay

Five of the most performing EPN strains (POP 139: *Steinernema carpocapsae*, POP 48: *S. feltiae*, POP 16: *Heterorhabditis bacteriophora*, POP 80: mix of *S. carpocapsae* and *H. bacteriophora*, and POP 138: mix of *S. carpocapsae* and *S. feltiae*) were tested against *P. japonica* 3rd instars in semi-field experiments. Based on the data collected in September 2018, the criteria used for the selection of the EPNs were, in order of importance, percentage of mortality, and median lethal times to 50% (LT50). Larvae of *P. japonica* were collected from the infested cornfield (pre-wintering 3rd instars) and maintained at 4 °C in native soil at the CREA-DC laboratory in Florence (Italy) until the time of the assay.

Larvae were acclimated individually in plastic cups, containing about 30 g of sterile soil and ryegrass seeds. Cups were incubated at 20 °C for 4 days before nematode inoculation. The selected EPN strains were reared and stored as above.

The experimental unit (*n* = 12 for each EPN) consisted of a plastic box (14.5 × 14.5 cm) with 400 g of sterile soil with about 13% humidity, determined by the gravimetric water content method [26], mixed with 2 g of ryegrass seeds and 4 *P. japonica* larvae at a 2 cm depth. A distilled water suspension of 1 mL containing about 5000 IJs (about 24 IJs/cm^2^) was inoculated into every box. Concerning the mixed isolates, the proportions of the two EPNs were as follows: 21% *S. carpocapsae*/79% *S. feltiae* for POP 138 and 87% *H. bacteriophora*/13% *S. carpocapsae* for POP 80. Only distilled water was added into the control (*n* = 12 boxes with 4 larvae each). Plastic boxes were stored at 23 °C in darkness for one week. Larval mortality was evaluated at the end of the experiment. Dead larvae were placed individually on modified white traps and observed daily to record the IJs emergence. Infective juveniles were collected and counted after 10 days to determine the progeny, which represents the reproductive potential.

### 2.5. Statistical Analysis

Data were first checked for normality and homogeneity of variance using Shapiro and Levene tests, respectively. When these assumptions were not met, non-parametric tests were used.

The habitat preference between the two *Steinernema* species was compared via contingency table analysis and the χ^2^ test. The preference between *S. carpocapsae* and *S. feltiae* isolates for the soil pH of the sampling sites was compared using the Welch *t*-test.

In laboratory assays, lethal times to 50% (LT50) were estimated by linear interpolation according to Marcus and Eaves [27]. The non-parametric Mann–Whitney test was used to compare the numbers of dead *P. japonica* larvae in May and September, and the pre-wintering larval mortality caused by the two *Steinernema* species, the LT50 between the two *Steinernema* species, and their emergence from cadavers. A t-test was used to compare the day of IJs emergence between *S. carpocapsae* and *S. feltiae* isolates. Larval mortality and progeny of the semi-field assay were analyzed using the Kruskal–Wallis test, followed by pairwise comparisons using the Wilcoxon rank-sum test with Benjamini and Hochberg’s correction.

Data were analyzed using R version 3.6.3 [28].

## 3. Results

### 3.1. Field Sampling and Isolation of Nematodes

Soil samples were collected in 155 selected areas trying to maintain a balance of representativeness between the two main habitats: woodlands and meadows with 77 samples and 72 samples, respectively. Six more areas were sampled in other habitats poorly represented in the ecosystem.

Natural EPNs were recovered from 39 out of the 155 soil samples collected (25.2%) (Table 1).

In particular, 36 samples contained only steinermatids (92.3%), 2 contained only heterorhabditids (5.1%), and 1 sample (2.6%) contained both genera. Among the isolated EPNs, the most abundant was *Steinernema carpocapsae* (Weiser) (Rhabditida: Steinernematidae) (48.7% of the total positive sample, *n* = 19), followed by *Steinernema feltiae* (Filipjev) (Rhabditida: Steinernematidae) (41%, *n* = 16). Concerning heterorhabditids, all the EPNs belonged to *Heterorhabditis bacteriophora* (Poinar) (Rhabditida: Heterorhabditidae) (5.1%, *n* = 2). Two samples were composed of two taxa: a mix of *H. bacteriophora* and *S. carpocapsae* and a mix of two species of *Steinernema*.

Most of the positive samples were recovered from perennial meadows and woodlands with a percentage of 51.3% and 38.5%, respectively. In particular, *H. bacteriophora* was present only in strongly acidic soils (pH 5.01–5.16) with sandy loam texture, collected only in open habitats (perennial meadows and uncultivated fields). *Steinernema carpocapsae* was mainly distributed in sites with extremely acidic–moderately acidic soil (pH 4.16–5.65) and sandy loam soil texture. It was more frequently recorded in open habitats, such as perennial meadows (78.9%), uncultivated soils with wild weeds and shrubs (10.5%), and in one cropland with alfalfa (5.3%). Only one *S. carpocapsae* strain (POP 139) was isolated from a woodland composed mainly of elm and black locust.

*Steinernema feltiae* was found associated with soils with sandy loam texture, pH ranging from extremely acidic to moderately acidic (3.71–5.78), and mainly closed habitat such as coniferous and deciduous woodland (81.3%). Most *S. feltiae* isolates (62.5%) were extracted from soils sampled in lowland mixed woods mainly composed of oak, black locust, hornbeam, hazel, and chestnut. One positive sample (POP 102) was isolated from soil collected from Norway spruce woodland and two *S. feltiae* strains were extracted from mixed coniferous/deciduous forests, in particular Scots pine/English oak (POP 91) and Scots pine/chestnut (POP 127). Instead, 18% of *S. feltiae* strains were isolated from perennial meadows.

Concerning the habitat preferences between the two *Steinernema*, *S. carpocapsae* was mainly related to open habitats, while *S. feltiae* to closed ones (χ^2^ = 17.85; df = 1; *p* < 0.0001). Further, the pH values of the habitats play a decisive role in the distribution of these two species: *S. feltiae* was found in more acidic soils (4.50 ± 0.17) in comparison to the number of *S. carpocapsae* (5.25 ± 0.06) (F = 18.658; df = 1, 18.322; *p* = 0.0003981).

### 3.2. Nematode Identification

Morphological and molecular analysis revealed that the EPN isolates matched with three described species: 21 *S. carpocapsae*, 17 *S. feltiae*, and 3 *H. bacteriophora*. Thirty-seven samples contained one strain, while two samples were composed of two EPNs associated with the following: POP 80 with *H. bacteriophora* and *S. carpocapsae*, and POP 138 with *S. carpocapsae* and *S. feltiae*. The morphological and morphometric data of nematodes were generally in agreement with the respective original description (Table S1).

Molecular identification based on the ITS sequence was performed on 117 individual nematodes coming from 39 POP isolates (three specimens/isolate). The amplification with TW81–AB28 primer pairs generated (at least) an amplicon in 37 out of the 39 isolations (94.9–96.0% successful amplifications). Comparison between the 96 obtained sequences led to identifying only three unique sequences. The BLAST homology search assigned the three sequences to as many different species: *H. bacteriophora* (MW226884), *S. carpocapse* (MW226882), and *S. feltiae* (MW226883).

Even if the analysis of large numbers of samples usually highlights the characteristic hypervariability of the ITS locus, the absence of nucleotide polymorphisms in the three species could be due to a very limited geographic provenance of the samples.

The ML tree based on the ITS locus clearly distinguished each *Steinernema* species, attributing them to a monophyletic origin. Moreover, the tree was able to univocally differentiate *S. carpocapse* from *S. feltiae* with a high bootstrap value (Figure S1).

### 3.3. Laboratory Virulence Assays

Concerning the laboratory assays, a significant difference was found among pre- and post-wintering grubs killed by EPNs (median May = 1, Q1 = 0, Q3 = 2 and median September = 4, Q1 = 2, Q3 = 5; W = 347; *n* = 78; *p* < 0.0001) (Table 2).

Considering only pre-wintering data, the percentages of insect mortality caused by EPNs varied between 0% and 100%. The greatest insect mortality (100%) was recorded for *H. bacteriophora* isolates (POP 9 and POP 16), the mix with *H. bacteriophora* and *S. carpocapsae* (POP 80), and one *S. carpocapsae* isolate (POP 139). Two *S. carpocapsae* isolates (POP 14 and POP 54) were avirulent towards *P. japonica* larvae, even if they killed *G. mellonella* larvae (data not shown). Between the two *Steinernema* species, *S. feltiae* caused significantly greater mortality than *S. carpocapsae* (median *S. feltiae* = 4, Q1 = 2.75, Q3 = 5 and median *S. carpocapsae* = 3, Q1 = 1, Q3 = 4; W = 93; *n* = 35; *p* = 0.04688); they emerged faster from the cadaver hosts (*S. feltiae*: 10.67 ± 0.58 and *S. carpocapsae*: 12.87 ± 0.76; t = 2.2945; df = 26.214; *p* = 0.03002) and from many larvae (median *S. feltiae* = 4, Q1 = 2, Q3 = 5 and median *S. carpocapsae* = 2, Q1 = 1, Q3 = 3; W = 56; *n* = 35; *p* = 0.01723). Instead, no significant difference was found in the time for killing the host, as indicated by LT50 (median *S. feltiae* = 9, Q1 = 5, Q3 = 10.375 and median *S. carpocapsae* = 8, Q1 = 7.25, Q3 = 9.50; W = 54; *n* = 35; *p* = 0.6876).

### 3.4. Semi-Field Virulence Assay

Among the 39 EPNs isolated, the five most performing taxa based on the features described above were as follows: POP 139 belonging to *S. carpocapsae*, POP 48 belonging to *S. feltiae*, POP 16 (*Heterorhabditis bacteriophora*), POP 80 (mix of *S. carpocapsae* 13% and *H. bacteriophora* 87%), and POP 138 (mix of *S. carpocapsae* 21% and *S. feltiae* 79%).

**Figure 2:**
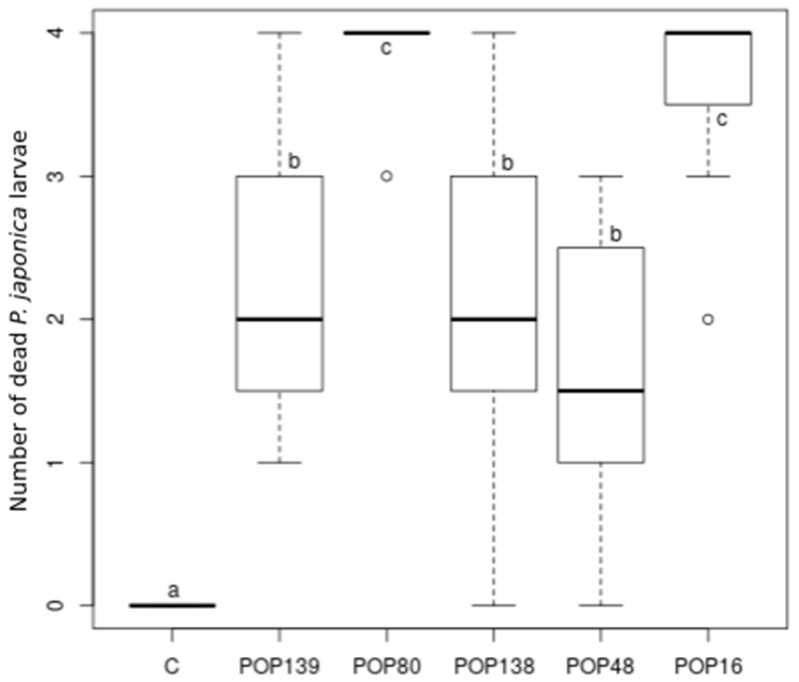
Number of *Popillia japonica* larvae killed by different entomopathogenic nematode isolates (C: control; POP 139: *Steinernema carpocapsae*; POP 80: *S. carpocapsae* + *Heterorhabditis bacteriophora;* POP 138: *S. carpocapsae* + *S. feltiae;* POP 48: *S. feltiae*; POP 16: *H. bacteriophora*). Different letters indicate significant differences among treatments.

There was a significant difference among the mortality caused by the five different EPNs and the control (Kruskal–Wallis χ^2^ = 52.596; df = 5; *p* < 0.0001) (Figure 2).

In particular, the isolates with *H. bacteriophora*, POP 80 and POP 16, caused the highest mortality, at 97.9% and 91.7%, respectively. Meanwhile, the isolates with *S. carpocapsae* and *S. feltiae* alone and mixed with each other killed a similar number of *P. japonica* larvae (54.2% POP 138 and POP 139, 39.6% POP 48) (Table 3).

**Figure 3:**
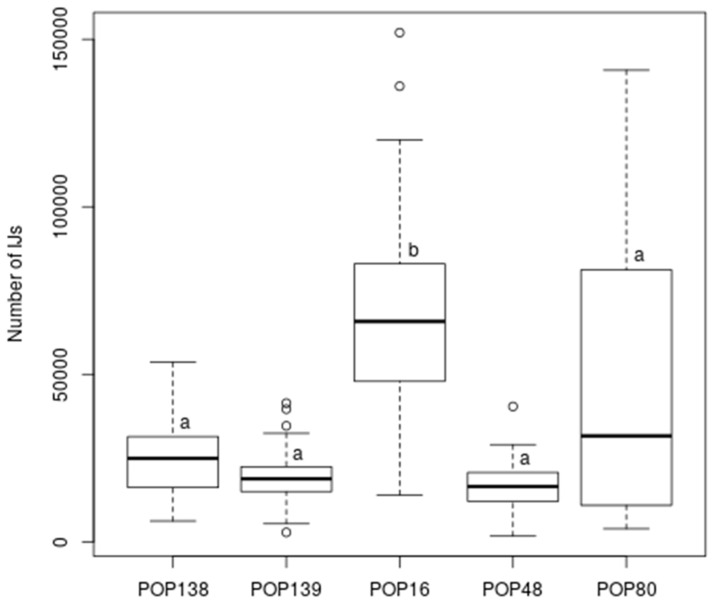
Number of infective juveniles (IJs) that emerged from *Popillia japonica* larvae killed by different entomopathogenic nematodes (POP 139: *Steinernema carpocapsae*; POP 80: *S. carpocapsae* + *Heterorhabditis bacteriophora;* POP 138: *S. carpocapsae* + *Steinernema feltiae;* POP 48: *S. feltiae*; POP 16: *H. bacteriophora*). Different letters indicate significant differences among EPN progenies.

Regarding progeny, there was a significant difference between the number of IJs produced by the five different EPNs (Kruskal–Wallis χ^2^ = 51.57; df = 4; *p* < 0.0001). In particular, the *H. bacteriophora* POP 16 reproduced more than the others, while no statistical differences were detected among POP 48, POP 80, POP 138, and POP 139 (Figure 3).

## 4. Discussion

In this study, the susceptibility of *Popillia japonica* larvae to indigenous EPN isolates, recovered from soil collected in the infested areas, was evaluated. The recovery frequency of 25.2% is higher than that reported in previous surveys on EPNs carried out in Italy, e.g., 5% [29], 6.5% [30], 14% [31], and 6% or 15.5% from pinewood or holm-oak wood habitats, respectively [32]. The EPNs’ occurrence in soil samples is highly variable in different surveys, with a recovery frequency ranging from less than 1% to more than 50% [33,34,35]. Our recovery frequency was similar to that reported in the Catalonia region of Spain (23.3%) by Garcia del Pino and Palomo [36] and in the USA (California, 26.3%, and Oregon, 23.7%) [37,38].

Steinernematids are usually recovered more often than heterorhabditids during non-targeted surveys [39]. This proportion is confirmed by our research, as well as by Tarasco et al. [30] in a previous study in Italy; however, it differs from the first survey on the biodiversity of EPNs in Italy, carried out in 1988 in the Emilia-Romagna region, where heterorhabditids formed 68% of the entomopathogenic nematodes [40].

All EPN isolates in this study were characterized at species level by morphological identification and confirmed by molecular analysis. The three species identified had already been discovered in the Italian territory [29,30,32,41]. Indeed, the Italian EPN fauna contains 12 described species: *Heterorhabditis bacteriophora*; *H. downesi* Stock, Griffin and Griffin et al.; *H. megidis* Poinar, Jackson and Klein; *Steinernema affine* (Bovien); *S. apuliae* Triggiani, Mráček, and Reid; *S. arenarium* (Artyukhovsky); *S. carpocapsae*; *S. feltiae*; *S. kraussei* (Steiner); *S. ichnusae* Tarasco, Mráček, Nguyen, and Triggiani; *S. vulcanicum* Clausi, Longo, Rappazzo, Tarasco, and Vinciguerra [30]; *Oscheius onirici* [42].

Moreover, two different EPN species were simultaneously extracted with the *Galleria mellonella* bait method from single soil samples. Several field surveys reported that steinernematid species often occur sympatrically (e.g., Stuart and Gaugler [43]; Campbell et al. [44]; Půža and Mráček [45]), and the co-occurrence of *S. carpocapsae* and *S. feltiae* had already been found by Garcia del Pino and Palomo [36]. On the contrary, concomitant steinernematid and heterorhabditid occurrences are less frequently reported [46,47]. Some laboratory assays showed the inter-specific interactions between different EPN species and their capability to produce mixed progeny inside the same cadaver [48,49,50,51]. Lewis et al. [52] asserted that species of *Steinernema* can penetrate and reproduce in the same insect host, though one species may suffer the effects of the competition more than the other one. This work confirmed this aspect of the two species of *Steinernema*, and also the co-existence of *S. carpocapsae* and *H. bacteriophora*. The proportion of progeny that emerged from *Galleria* larvae in the Kock’s postulates assay was similar in both cases (POP 138: 45% *S. carpocapsae*/55% *S. feltiae* and POP 80: 61% *H. bacteriophora*/39% *S. carpocapsae*); however, after storage and the multiplication of EPNs in *G. mellonella* larvae to obtain fresh progeny for the semi-field assay, the presence of *S. carpocapsae* IJs decreased in both cases, at 21% for POP 138 and 13% for POP 80. Moreover, these percentages reached very low values in the progeny that emerged from *P. japonica* larvae that died in the semi-field assay (3.7% in POP 80; 8.8% in POP 138).

The EPN occurrence is influenced by the soil characteristics, especially by the texture [53]. Most studies report that EPNs are more prevalent in soils with a high sand content, which promotes the mobility and survival of nematodes [37,54,55]. Indeed, in this study, 97.4% of the sampled soils had a sandy loam texture, typical of the areas along the Ticino river, and only one *S. carpocapsae* isolate (POP 44) was extracted from an area characterized by silty soil texture.

Another important soil parameter that influenced the EPN occurrence is the pH [38]. It is commonly reported that EPNs were found in soils with a wide spectrum of pH, from acidic (pH 4) to alkaline (pH 8). Mwaniki et al. [56] suggested that steinernematids are more suited to pH < 6 and heterorhabditids to pH > 6. Since all sampled soils in the study area were characterized by low pH [22,23], this can explain the low occurrence of *Heterorhabditis* in our sampling area where only three *H. bacteriophora* isolates were collected from soils with a strongly acidic pH. All the *Steinernema* isolates were extracted from soils with a pH < 6, and four *S. feltiae* isolates (POP 101, POP 127, POP 152, and POP 153) from the soil with a pH < 4. Only a few works reported EPNs found in soil with a pH < 4 [57,58].

In this study, different EPN species showed a distinct habitat preference. Open habitats, such as perennial meadows, cropland, and uncultivated fields, were suitable for *S. carpocapsae*, compared to *S. feltiae* which has been found mainly in closed habitats (woodland). The literature on habitat preference for EPNs is various and contradictory. In some cases, soil samples collected from agricultural lands had more EPNs than those from natural habitats [36,59,60,61]. However, opposing results have also been found with EPNs more prevalent in natural habitats than agricultural ones [62,63,64]. In this survey, EPNs were found in both habitats, even if perennial meadows, generally considered agricultural habitats, are not subject to intense management which can alter the edaphic conditions through soil tillage, exposing EPNs to unfavorable abiotic and biotic conditions.

Akhurst and Bedding [65] suggested that the abundance of suitable insect hosts seems to be crucial for EPN occurrence and distribution. Therefore, both forests and open habitats can host different EPN species, and they are generally more frequent in insect-rich habitats. In this survey, perennial meadows and uncultivated fields presented a high number of *P. japonica* larvae (45 ± 14 larvae/m^2^). In woodlands, however, no larvae of this pest were found, but it is known that coniferous and deciduous forests are rich in insects which pupate in the soil, creating an ideal environment for the persistence of EPNs [66,67].

The laboratory assays carried out with EPN strains isolated from our study area showed that there was a significant difference in the mortality of larvae collected in the two different periods. Paoli et al. [25] reported that *P. japonica* 3rd-instar larvae were more susceptible to *H. bacteriophora* in the pre-wintering than in the post-wintering period. This work not only confirmed the different susceptibility of *P. japonica* larvae to *H. bacteriophora*, but also to steinernematids.

The 39 isolates were all highly virulent to *G. mellonella* larvae, producing 83.3–100% larval mortality in 24–72 h after 31 JIs/cm^2^ exposition (data not shown), but high intraspecific variability in the percentage mortality of *P. japonica* larvae was shown for both steinernematids recovered. These differences among strains of *S. carpocapsae* and *S. feltiae* had also been previously reported by Simões et al. [68] and Tarasco [69], against *G. mellonella*. The isolates containing *H. bacteriophora* alone or mixed with steinernematids were the most virulent ones and confirmed the results shown by Marianelli et al. [13].

## 5. Conclusions

This study of the occurrence and the pathogenicity of different EPN strains in this geographical area infested by *P. japonica* indicated that soils in the Ticino valley are rich in EPNs. Moreover, the nematodes isolated during this survey could be used in biological control programs against this dangerous alien pest, since native isolates of EPNs possess physiological traits that are adapted to local ecological conditions.

The idea of supporting the ecosystem by amplifying the entomopathogenic fauna that thrives in its native soil could be considered the basis for an eco-friendly approach against alien pests.

## Figures and Tables

**Table 1:** Natural distribution of entomopathogenic nematodes in *Popillia japonica*-infested areas in the Piedmont region.

EPN Species	Soil Sample	Habitat	Soil Type	pH	Geographical Coordinates WGS 84
*Steinernema carpocapsae*	POP 4	Perennial meadows	Sandy loam	5.03	45°29′37″ N; 8°44′24″ E
	POP 5	Perennial meadows	Sandy loam	5.31	45°29′59″ N; 8°43′40″ E
	POP 6	Perennial meadows	Sandy loam	5.15	45°30′2″ N; 8°43′34″ E
	POP 8	Perennial meadows	Sandy loam	5.50	45°18′7″ N; 8°44′26″ E
	POP 12	Perennial meadows	Sandy loam	5.24	45°29′18″ N; 8°44′43″ E
	POP 14	Perennial meadows	Sandy loam	5.20	45°29′7″ N; 8°45′39″ E
	POP 28	Cropland	Sandy loam	4.96	45°27′36″ N; 8°47′15″ E
	POP 34	Perennial meadows	Sandy loam	5.32	45°24′9″ N; 8°49′3″ E
	POP 38	Perennial meadows	Sandy loam	5.55	45°28′55″ N; 8°46′27″ E
	POP 44	Perennial meadows	Silty loam	5.05	45°35′8″ N; 8°38′2″ E
	POP 46	Perennial meadows	Sandy loam	5.33	45°40′10″ N; 8°38′55″ E
	POP 54	Perennial meadows	Sandy loam	5.31	45°36′25″ N; 8°38′48″ E
	POP 55	Perennial meadows	Sandy loam	5.65	45°36′48″ N; 8°39′36″ E
	POP 59	Perennial meadows	Sandy loam	5.61	45°36′7″ N; 8°39′40″ E
	POP 69	Perennial meadows	Sandy loam	5.10	45°29′39″ N; 8°44′56″ E
	POP 70	Uncultivated field	Sandy loam	5.08	45°29′26″ N; 8°44′54″ E
	POP 71	Uncultivated field	Sandy loam	5.17	45°29′27″ N; 8°44′44″ E
	POP 74	Perennial meadows	Sandy loam	5.51	45°29′25″ N; 8°44′34″ E
	POP 139	Woodland	Sandy loam	4.73	45°29′51″ N; 8°44′20″ E
*Steinernema feltiae*	POP 48	Woodland	Sandy loam	4.65	45°40′10″ N; 8°38′48″ E
	POP 73	Perennial meadows	Sandy loam	5.26	45°29′30″ N; 8°44′39″ E
	POP 78	Perennial meadows	Sandy loam	5.78	45°29′15″ N; 8°45′37″ E
	POP 79	Perennial meadows	Sandy loam	5.61	45°29′14″ N; 8°45′27″ E
	POP 91	Woodland	Sandy loam	4.19	45°37′0″ N; 8°40′45″ E
	POP 100	Woodland	Sandy loam	4.08	45°39′5″ N; 8°39′35″ E
	POP 101	Woodland	Sandy loam	3.95	45°39′3″ N; 8°39′22″ E
	POP 102	Woodland	Sandy loam	5.29	45°39′12″ N; 8°39′17″ E
	POP 103	Woodland	Sandy loam	4.11	45°39′26″ N; 8°39′5″ E
	POP 105	Woodland	Sandy loam	4.25	45°40′4″ N; 8°39′11″ E
	POP 127	Woodland	Sandy loam	3.71	45°31′24″ N; 8°42′12″ E
	POP 135	Woodland	Sandy loam	4.54	45°30′9″ N; 8°43′25″ E
	POP 142	Woodland	Sandy loam	4.74	45°29′34″ N; 8°45′41″ E
	POP 144	Woodland	Sandy loam	4.12	45°29′11″ N; 8°45′51″ E
	POP 152	Woodland	Sandy loam	3.95	45°28′14″ N; 8°45′52″ E
	POP 153	Woodland	Sandy loam	3.74	45°27′59″ N; 8°46′14″ E
*Heterorhabditis bacteriophora*	POP 9	Perennial meadows	Sandy loam	5.16	45°29′26″ N; 8°44′33″ E
	POP 16	Uncultivated field	Sandy loam	5.01	45°29′4″ N; 8°45′45″ E
*H. bacteriophora + S. carpocapsae*	POP 80	Perennial meadows	Sandy loam	5.09	45°29′15″ N; 8°45′40″ E
*S. carpocapsae + S. feltiae*	POP 138	Woodland	Sandy loam	4.16	45°29′55″ N; 8°44′16″ E

**Table 2:** Results of laboratory assays. Percentage of mortality, lethal times to 50% (LT50), and days and percentage of infective juveniles (IJs) emergence of all isolates against *Popillia japonica* larvae in pre- (May) and post-wintering (September) periods.

EPN Species	Soil Sample	Mortality (%)		LT50		Days of Emergence (Mean)		Emergence (%)	
		May	September	May	September	May	September	May	September
	Control	0.0	0.0						
*Steinernema carpocapsae*	POP 4	16.7	50.0	-	8.0	-	13	-	33.3
	POP 5	16.7	50.0	-	7.0	14	13	16.7	50.0
	POP 6	16.7	83.3	-	4.0	-	9	-	50.0
	POP 8	33.3	16.7	-	-	8	15	16.7	16.7
	POP 12	0.0	33.3	-	-	-	15	-	16.7
	POP 14	0.0	0.0	-	-	-	-	-	-
	POP 28	33.3	66.7	-	8.0	15	15	16.7	50.0
	POP 34	33.3	33.3	-	-	10	13	33.3	16.7
	POP 38	33.3	16.7	-	-	13	19		16.7
	POP 44	0.0	66.7	-	10.0	-	13	-	50.0
	POP 46	0.0	16.7	-	-	-	10	-	16.7
	POP 54	0.0	0.0	-	-	-	-	-	-
	POP 55	50.0	50.0	9.0	8.0	6	9	50.0	16.7
	POP 59	50.0	66.7	6.0	8.0	19	11	50.0	50.0
	POP 69	33.3	33.3	-	-	-	-	-	-
	POP 70	0.0	16.7	-	-	-	-	-	-
	POP 71	16.7	50.0	-	10.0	-	11	-	50.0
	POP 74	0.0	66.7	-	11.0	-	17	-	33.3
	POP 139	16.7	100.0	-	6.0	-	10	-	100.0
*Steinernema feltiae*	POP 48	33.3	83.3	-	2.6	11	14	33.3	50.0
	POP 73	33.3	83.3	-	3.0	22	12	33.3	83.3
	POP 78	16.7	83.3	-	10.0	11	12	16.7	83.3
	POP 79	16.7	0.0	-	-	-	-	-	-
	POP 91	16.7	83.3	-	11.5	15	9	16.7	83.3
	POP 100	33.3	66.7	-	5.0	19	15	33.3	50.0
	POP 101	0.0	33.3	-	-	-	13	-	33.3
	POP 102	16.7	66.7	-	8.0	9	11	16.7	66.7
	POP 103	33.3	83.3	-	8.0	11	11	16.7	83.3
	POP 105	33.3	33.3	-	-	-	11	-	33.3
	POP 127	33.3	66.7	-	5.0	12	9	16.7	33.3
	POP 135	16.7	16.7	-	-	-	8	-	16.7
	POP 142	0.0	66.7	-	12.0	-	9	-	66.7
	POP 144	50.0	66.7	13	12.0	12	9	33.3	66.7
	POP 152	0.0	83.3	-	10.0	-	10	-	83.3
	POP 153	16.7	50.0	-	10.0	-	7	-	50.0
*Heterorhabditis bacteriophora*	POP 9	100.0	100.0	7.5	8.0	14	13	83.3	100.0
	POP 16	83.3	100.0	6.0	4.7	12	11	83.3	100.0
*H. bacteriophora + S. carpocapsae*	POP 80	83.3	100.0	5.5	2.8	16	13	66.7	83.3
*S. carpocapsae + S. feltiae*	POP 138	33.3	83.3	-	3.0	8	9	33.3	83.3

**Table 3:** *Popillia japonica* mortality caused by the five most performing entomopathogenic nematodes (EPN) isolates and their progeny in the semi-field assay. *H.b.*: *Heterorhabditis bacteriophora*; *S.f*.: *Steinernema feltiae*; *S.c*.: *Steinernema carpocapsae.*

Sample	Nematode	Mortality (%)	Progeny (± Standard Error)		
Control		0		-	
POP16	*H.b.*	91.7	68,339	±	4639.71
POP48	*S.f.*	39.6	17,710	±	2046.30
POP80	*S.c. + H.b.*	97.9	44,964	±	5983.27
POP138	*S.c. + S.f.*	54.2	25,729	±	2590.45
POP139	*S c.*	54.2	20,173	±	1847.10

## Supplementary Materials

The following are available online at https://www.mdpi.com/2075-4450/11/11/804/s1. Table S1: Morphometric characters of entomopathogenic nematode isolates, Figure S1: Maximum Likelihood phylogenetical reconstruction based on ITS locus.
