# Supplementary material for: Evaluation of Indigenous Entomopathogenic Nematodes as Potential Biocontrol Agents against Popillia japonica (Coleoptera: Scarabaeidae) in Northern Italy
Source: Insects. 2020 Nov 14;11(11):804. doi: 10.3390/insects11110804 (PMC7697182; doi:10.3390/insects11110804)
Supplement: Supplementary file 1 [file insects-11-00804-s001.pdf]

Table S1. Morphometric characters of entomopathogenic nematode isolates. All measurements are in μm: mean ± SD (range).

Isolate	L	ES	EP	T	a	b	c	D%	E%
Steinernema carpocapsae									
POP 4	575.77 ± 9.9 (559.8-586.6)	118.6 ± 3.5 (110.1-123.0)	40.7 ± 1.0 (38.5-42.1)	54.1 ± 3.2 (50.1-60.8)	22.1 ± 0.6 (21.0-23.0)	4.9 ± 0.1 (4.8-5.1)	10.7 ± 0.6 (9.6-11.6)	34.3 ± 0.6 (33.4-35.5)	75.4 ± 3.9 (67.1-80.8)
POP 5	602.0 ± 14.8 (580.1-621.9)	110.9 ± 4.5 (105.9-119.4)	33.8 ± 2.3 (31.0-38.0)	57.1 ± 4.5 (49.4-63.8)	19.0 ± 2.1 (16.0-21.2)	5.4 ± 0.3 (5.0-5.8)	10.6 ± 0.7 (9.6-12.0)	30.5 ± 2.0 (27.2-33.8)	59.6 ± 6.8 (52.5-70.9)
POP 6	586.4 ± 20.4 (542.5-607.2)	108.3 ± 7.0 (97.3-116.3)	33.8 ± 3.9 (29.5-38.9)	52.2 ± 3.3 (47.3-57.0)	21.5 ± 1.2 (19.0-23.1)	5.4 ± 0.3 (5.0-5.9)	11.3 ± 0.5 (10.7-12.4)	31.9 ± 2.4 (28.6-35.0)	64.8 ± 5.2 (56.7-70.3)
POP 8	516.7 ± 35.9 (460.9-560.1)	117.1 ± 6.0 (109.0-125.3)	33.1 ± 2.9 (28.0-37.6)	49.4 ± 4.0 (43.1-56.7)	22.8 ± 1.8 (19.9-25.2)	4.4 ± 0.4 (3.7-4.9)	10.5 ± 0.7 (9.6-11.8)	28.3 ± 2.6 (23.0-32.4)	67.3 ± 7.2 (55.0-77.5)
POP 12	596.2 ± 18.8 (566.4-614.1)	128.2 ± 6.4 (121.3-137.9)	37.0 ± 3.2 (32.0-42.0)	54.9 ± 3.3 (49.8-59.6)	21.8 ± 1.7 (19.5-23.8)	4.7 ± 0.2 (4.2-5.0)	10.9 ± 0.7 (10.1-12.0)	28.9 ± 2.3 (23.6-31.3)	67.4 ± 3.7 (62.2-72.8)
POP 14	587.9 ± 21.9 (552.8-629.3)	151.9 ± 14.2 (125.0-170.5)	34.9 ± 2.5 (31.2-38.0)	54.7 ± 2.8 (50.1-58.3)	23.2 ± 0.9 (22.0-24.8)	3.9 ± 0.3 (3.5-4.4)	10.8 ± 0.6 (10.0-11.4)	23.2 ± 3.1 (18.8-28.1)	63.9 ± 4.6 (57.0-70.9)
POP 28	558.6 ± 22.4 (522.0-585.4)	105.5 ± 6.1 (96.3-115.9)	33.6 ± 1.4 (31.3-35.7)	57.7 ± 5.5 (51.9-69.8)	18.1 ± 1.4 (16.7-20.8)	5.3 ± 0.2 (4.9-5.5)	9.8 ± 1.0 (8.2-11.3)	31.9 ± 1.8 (29.4-34.0)	58.6 ± 4.8 (50.3-64.0)
POP 34	560.4 ± 33.8 (516.8-617.5)	130.9 ± 9.9 (116.4-144.7)	37.6 ± 9.9 (31.9-40.9)	51.2 ± 2.4 (47.7-54.2)	20.8 ± 1.8 (18.2-23.4)	4.3 ± 0.2 (4.0-4.5)	10.9 ± 0.4 (10.4-11.5)	28.8 ± 1.5 (27.0-30.8)	73.5 ± 4.2 (64.3-77.3)
POP 38	575.1 ± 43.5 (516.0-659.1)	120.6 ± 8.6 (103.1-129.5)	36.5 ± 3.9 (30.1-41.3)	53.6 ± 5.1 (42.1-59.2)	21.5 ± 2.4 (19.4-27.4)	4.8 ± 0.3 (4.3-5.3)	10.8 ± 0.9 (9.7-12.3)	30.3 ± 2.5 (26.2-34.4)	68.4 ± 6.4 (54.4-77.2)
POP 44	514.4 ± 26.3 (483.3-569.9)	123.2 ± 7.9 (109.6-133.8)	33.1 ± 2.5 (29.7-36.6)	51.6 ± 3.1 (47.1-55.4)	20.1 ± 1.5 (18.4-22.9)	4.2 ± 0.1 (3.9-4.4)	10.0 ± 0.5 (9.0-10.6)	26.9 ± 2.0 (23.8-29.8)	64.1 ± 4.2 (57.0-70.0)
POP 46	582.5 ± 10.3 (562.7-596.8)	125.4 ± 3.4 (120.8-130.1)	40.3 ± 1.5 (37.8-42.5)	59.9 ± 2.5 (54.7-64.1)	21.7 ± 1.4 (19.3-23.5)	4.6 ± 0.2 (4.5-4.9)	9.7 ± 0.4 (9.2-10.6)	32.1 ± 1.2 (29.8-33.7)	67.3 ± 2.7 (62.7-71.1)
POP 54	595.1 ± 29.4 (528.8-631.4)	126.3 ± 7.4 (117.3-138.2)	37.4 ± 4.0 (28.7-41.6)	56.9 ± 5.3 (50.4-66.2)	17.2 ± 0.8 (16.1-18.4)	4.7 ± 0.3 (4.4-5.4)	10.5 ± 1.0 (9.0-11.8)	29.5 ± 2.4 (24.5-32.4)	66.6 ± 11.4 (43.4-78.0)
POP 55	574.2 ± 22.2 (550.8-626.5)	113.2 ± 13.4 (88.7-124.6)	37.7 ± 1.9 (35.3-41.2)	56.4 ± 3.4 (51.1-61.2)	19.8 ± 1.7 (18.3-24.4)	5.1 ± 0.7 (4.5-6.3)	10.2 ± 0.7 (9.2-11.3)	33.9 ± 6.1 (28.3-46.2)	66.8 ± 2.7 (62.1-72.4)
POP 59	550.7 ± 21.0 (510.8-585.3)	137.5 ± 11.1 (118.1-151.0)	40.9 ± 5.0 (36.3-48.3)	54.1 ± 3.7 (46.7-58.3)	20.6 ± 1.8 (17.9-23.1)	4.0 ± 0.3 (3.7-4.7)	10.2 ± 0.7 (9.3-11.8)	29.8 ± 3.1 (26.3-33.4)	76.6 ± 8.4 (66.7-89.6)
POP 69	589.3 ± 20.6 (550.5-620.4)	131.5 ± 8.1 (122.7-148.1)	35.4 ± 3.3 (28.5-40.0)	55.3 ± 4.6 (49.7-65.0)	27.5 ± 1.4 (19.5-22.8)	4.5 ± 0.2 (4.1-4.7)	10.7 ± 0.9 (9.3-12.2)	26.9 ± 2.3 (21.9-30.6)	64.1 ± 6.5 (55.2-72.8)
POP 70	486.2 ± 55.3 (412.6-550.6)	119.7 ± 5.4 (109.9-128.4)	34.4 ± 2.7 (31.3-39.2)	52.5 ± 3.7 (46.0-57.6)	20.4 ± 2.5 (17.9-25.2)	4.1 ± 0.4 (3.4-4.5)	9.3 ± 1.0 (8.2-11.4)	28.8 ± 2.4 (26.1-33.1)	65.6 ± 2.8 (60.7-68.3)
POP 71	517.9 ± 25.0 (481.0-553.8)	128.2 ± 6.6 (117.1-138.8)	36.1 ± 1.2 (34.2-38.2)	49.8 ± 2.8 (47.2-55.4)	21.3 ± 0.8 (20.2-22.6)	4.0 ± 0.2 (3.8-4.5)	10.4 ± 0.6 (9.2-11.2)	28.2 ± 1.1 (26.5-29.3)	72.7 ± 4.5 (66.1-79.3)
POP 74	526.2 ± 31.2 (473.9-579.7)	137.1 ± 9.8 (127.0-152.4)	37.5 ± 2.4 (35.3-42.1)	53.8 ± 2.9 (48.0-57.0)	20.6 ± 1.3 (18.2-22.3)	3.9 ± 0.3 (3.4-4.3)	9.8 ± 0.8 (8.5-11.1)	27.4 ± 1.5 (24.3-30.2)	69.8 ± 4.6 (64.0-74.9)
POP 80	514.9 ± 44.1 (448.5-589.7)	118.3 ± 10.4 (102.8-136.2)	30.8 ± 4.1 (25.4-39.2)	53.2 ± 3.4 (47.3-59.1)	19.9 ± 2.0 (22.8-29.5)	4.4 ± 0,3 (3.9-4.7)	9.7 ± 0.6 (8.9-10.5)	26.1 ± 2.6 (22.9-29.5)	57.9 ± 6.3 (48.4-67.8)
POP 138	501.8 ± 20.9 (477.8-546.1)	109.0 ± 6.7 (99.6-117.9)	31.0 ± 3.5 26.6-36.7)	50.7 ± 2.8 (45.6-54.6)	18.8 ± 2.0 (16.4-20.6)	4.6 ± 0.2 (4.2-5.0)	9.9 ± 0.5 (9.2-10.8)	28.4 ± 1.7 (25.7-31.1)	75.6 ± 5.1 (55.1-69.1)
POP 139	580.8 ± 19.6 (547.1-606.8)	131.9 ± 5.9 (123.2-142.8)	34.4 ± 2.6 (30.4-37.5)	56.6 ± 2.8 (51.2-59.6)	23.2 ± 1.3 (21.7-25.7)	4.4 ± 0.1 (4.2-4.5)	10.3 ± 0.3 (9.9-10.8)	26.1 ± 2.0 (23.0-29.3)	60.9 ± 5.1 (52.5-67.5)
Steinernema feltiae									
POP 48	734.5 ± 24.5 (690.2-784.5)	105.0 ± 2.4 (101.0-109.0)	53.4 ± 3.7 (47.0-59.7)	74.4 ± 3.8 (66.6-79.2)	27.6 ± 0.9 (25.6-29.1)	7.0 ± 0.2 (6.7-7.6)	9.9 ± 0.3 (9.4-10.4)	50.9 ± 3.5 (46.5-57.7)	71.8 ± 3.6 (66.3-77.1)
POP 73	881.2 ± 48.2 (810.6-967.2)	139.2 ± 7.6 (128.7-152.2)	64.2 ± 4.5 (55.2-71.6)	83.0 ± 5.0 (78.0-93.2)	23.7 ± 1.3 (21.6-25.6)	6.3 ± 0.3 (5.9-6.9)	10.6 ± 0.6 (9.9-11.9)	46.1 ± 2.4 (41.2-48.5)	77.6 ± 7.3 (65.5-86.7)
POP 78	853.5 ± 43.9 (775.1-912.6)	139.3 ± 7.2 (125.2-149.2)	64.0 ± 4.5 (54.6-70.2)	77.9 ± 6.0 (68.8-88.1)	27.2 ± 2.4 (23.9-31.1)	6.1 ± 0.3 (5.8-6.7)	11.0 ± 0.6 (9.9-11.6)	46.0 ± 2.9 (40.5-49.5)	82.5 ± 7.1 (71.8-90.0)
POP 79	862.8 ± 30.6	126.8 ± 3.6	60.0 ± 1.7	76.6 ± 3.3	28.3 ± 1.2	6.8 ± 0.3	11.3 ± 0.4	47.3 ± 2.3	78.3 ± 3.2

	(814.0-907.9)	(122.3-133.3)	(57.2-62.5)	(69.1-80.0)	(26.1-30.3)	(6.5-7.4)	(10.6-11.8)	(44.6-51.1)	(74.5-82.8)
POP 91	862.0 ± 45.7 (798.9-939.1)	131.6 ± 5.0 (122.4-140.7)	59.7 ± 3.2 (53.4-63.5)	81.0 ± 6.1 (72.8-89.0)	26.9 ± 1.5 (24.9-28.9)	6.6 ± 0.4 (6.1-7.3)	10.7 ± 0.5 (10.0-11.4)	45.4 ± 1.7 (42.8-47.5)	73.9 ± 5.3 (67.9-82.9)
POP 100	860.8 ± 32.2 (805.9-903.0)	146.1 ± 6.3 (134.0-152.5)	60.9 ± 3.2 (55.0-65.0)	86.3 ± 7.2 (73.5-97.0)	26.2 ± 1.0 (24.7-27.39	5.9 ± 0.3 (5.6-6.6)	10.0 ± 0.8 (9.0-11.6)	41.7 ± 2.4 (38.4-46.8)	70.8 ± 4.9 (65.5-80.6)
POP 101	823.2 ± 43.8 (752.3-890.3)	129.8 ± 4.6 (121.1-135.9)	58.3 ± 2.2 (54.3-61.0)	79.4 ± 4.3 (71.9-87.2)	26.5 ± 1.3 (25.2-29.5)	6.3 ± 0.3 (5.9-6.7)	10.4 ± 0.5 (9.6-11.3)	45.0 ± 2.0 (41.9-48.6)	73.6 ± 3.5 (69.5-78.9)
POP 102	788.8 ± 27.2 (751.2-827.5)	127.1 ± 7.8 (117.3-139.0)	59.6 ± 4.2 (52.5-65.0)	81.7 ± 3.9 (74.7-88.4)	27.6 ± 1.9 (24.6-30.4)	6.2 ± 0.4 (5.9-6.8)	9.7 ± 0.5 (9.0-10.4)	47.1 ± 4.9 (40.6-55.4)	73.1 ± 6.3 (63.3-82.1)
POP 103	844.0 ± 39.4 (791.0-911.4)	127.7 ± 2.9 (124.6-132.4)	61.1 ± 1.9 (59.2-65.0)	83.0 ± 4.7 (75.8-89.8)	28.3 ± 2.2 (23.0-29.9)	6.6 ± 0.3 (6.3-7.3)	10.2 ± 0.5 (9.7-11.4)	47.8 ± 0.8 (47.1-49.7)	73.8 ± 4.5 (65.9-82.5)
POP 105	887.6 ± 24.7 (837.6-922.9)	135.5 ± 4.8 (128.5-144.6)	61.4 ± 3.7 (53.6-65.0)	86.0 ± 2.6 (80.8-90.2)	28.3 ± 2.0 (24.2-30.5)	6.6 ± 0.3 (6.0-7.0)	10.3 ± 0.3 (9.9-10.7)	45.3 ± 2.6 (41.3-48.9)	71.4 ± 3.9 (61.8-75.7)
POP 127	851.8 ± 40.9 (797.2-911.5)	135.2 ± 5.8 (127.5-146.3)	57.8 ± 3.4 (51.3-62.6)	78.4 ± 4.5 (72.7-87.9)	27.4 ± 1.5 (25.1-29.2)	6.3 ± 0.2 (6.0-6.9)	10.9 ± 0.4 (10.0-11.4)	42.7 ± 2.1 (39.0-45.7)	73.9 ± 4.0 (65.4-78.8)
POP 135	873.9 ± 22.4 (844.7-918.4)	139.0 ± 6.1 (127.8-149.7)	63.1 ± 1.4 (61.4-65.6)	81.9 ± 5.0 (76.7-92.2)	27.2 ± 1.5 (24.8-29.6)	6.3 ± 0.3 (5.7-6.9)	10.7 ± 0.6 (9.4-11.2)	45.5 ± 2.4 (42.2-50.2)	77.2 ± 4.1 (69.7-81.2)
POP 138	761.3 ± 24.8 (714.8-800.5)	116.1 ± 4.4 (110.0-125.1)	57.2 ± 1.9 (54.6-59.9)	75.6 ± 2.1 (72.0-78.4)	27.3 ± 2.0 (23.6-30.1)	6.6 ± 0.2 (6.2-6.9)	10.1 ± 0.3 (9.6-10.4)	49.3 ± 0.9 (47.1-50.2)	75.6 ± 3.3 (70.8-80.3)
POP 142	854.1 ± 55.1 (725.8-925.0)	129.4 ± 4.3 (121.9-135.5)	59.7 ± 3.2 (54.0-63.4)	77.6 ± 5.5 (65.2-83.4)	26.2 ± 1.0 (24.3-27.5)	6.6 ± 0.3 (6.0-7.1)	11.0 ± 0.5 (10.3-11.5)	46.1 ± 2.0 (42.5-49.0)	77.1 ± 5.2 (66.5-82.8)
POP 152	781.5 ± 32.7 (732.7-837.9)	136.5 ± 5.7 (126.7-142.7)	62.3 ± 1.5 (60.3-65.8)	78.2 ± 4.5 (68.5-83.3)	26.9 ± 1.9 (25.0-30.1)	5.7 ± 0.4 (5.2-6.5)	10.0 ± 0.4 (9.3-10.7)	45.7 ± 1.9 (43.6-48.8)	79.8 ± 5.0 (74.4-90.5)
POP 153	876.7 ± 37.8 (826.9-954.8)	131.3 ± 10.4 (122.3-158.2)	64.0 ± 5.9 (57.5-75.0)	78.9 ± 3.9 (74.6-85.3)	26.0 ± 2.1 (23.8-30.1)	6.7 ± 0.4 (6.0-7.3)	11.1 ± 0.6 (10.0-12.2)	48.8 ± 3.0 (45.1-55.3)	81.3 ± 7.4 (72.1-94.3)
					Heterorhabditis bacteriophora				
POP 9	604.6 ± 24.1 (570.0-649.0)	125.8 ± 5.2 (115.4-131.5)	100.8 ± 2.8 (95.6-105.5)	93.9 ± 4.6 (85.2-98.6)	24.7 ± 0.7 (23.6-25.8)	4.8 ± 0.2 (4.6-5.0)	6.4 ± 0.3 (6.0-6.9)	80.2 ± 3.5 (76.6-86.5)	107.6 ± 5.1 (102.5-120.2)
POP 16	577.3 ± 14.3 (556.8-600.6)	128.0 ± 4.2 (122.6-134.2)	99.5 ± 1.5 (97.5-102.1)	93.6 ± 4.2 (85.6-98.8)	24.4 ± 1.5 (21.1-25.9)	4.5 ± 0.1 (4.3-4.7)	6.2 ± 0.2 (5.8-6.5)	77.8 ± 2.3 (73.5-80.3)	106.4 ± 5.0 (99.9-115.7)
POP 80	542.5 ± 21.8 (503.9-572.2)	119.3 ± 5.7 (110.9-130.7)	103.3 ± 7.9 (93.1-114.7)	86.7 ± 4.7 (78.8-94.5)	19.5 ± 1.2 (17.8-21.9)	4.6 ± 0.3 (4.0-4.8)	6.3 ± 0.4 (5.7-6.9)	86.7 ± 7.5 (80.1-98.7)	119.5 ± 10.9 (100.5-130.7)

L: Body length; ES: distance from anterior end to base of oesophagus; EP: distance from anterior end to excretory pore (EP); T: tail length; a: L/maximum boy diameter; b: L/ES; c: L/T; D%: EP/ES x 100; E%: EP/T x 100.

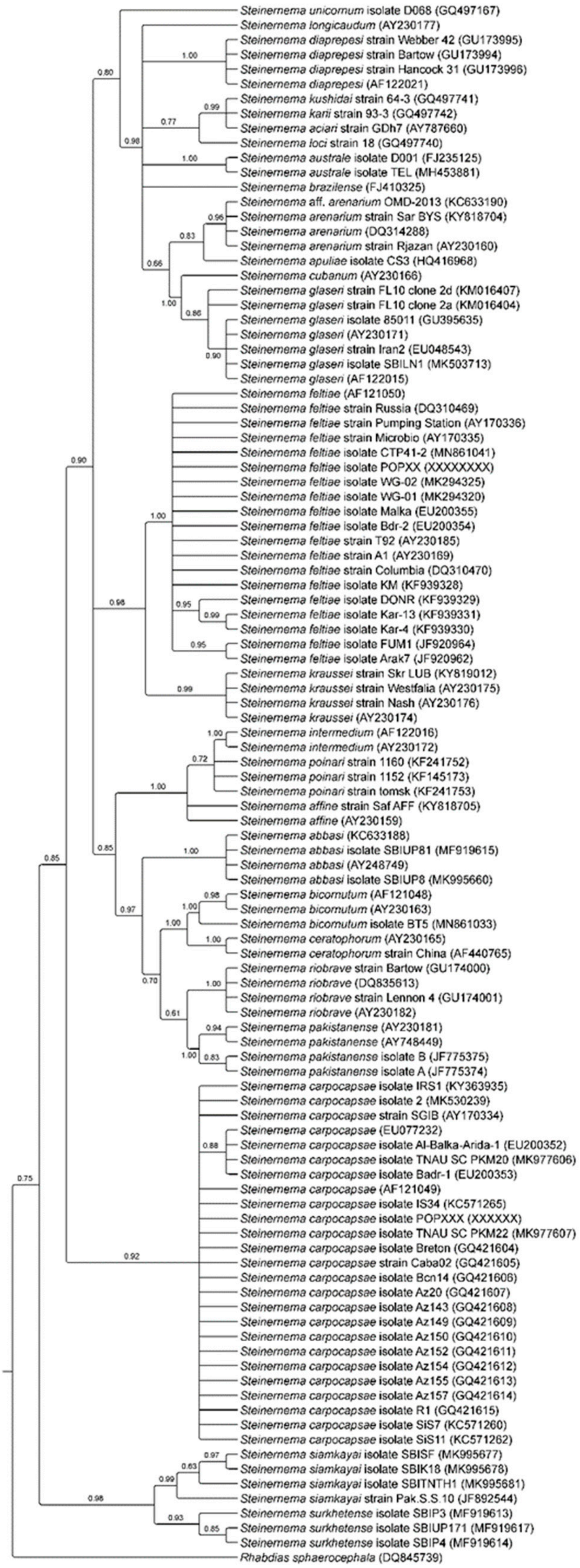

Figure S1. Maximum Likelihood phylogenetical reconstruction based on ITS locus. The tree is displayed. Accession numbers of each leaf were annotated in brackets. The tree was displayed in a condensed form with a 50% cut-off on bootstrap support, values were reported near each node.
